# Detection of cell-free foetal DNA fraction in female-foetus bearing pregnancies using X-chromosomal insertion/deletion polymorphisms examined by digital droplet PCR

**DOI:** 10.1038/s41598-020-77084-0

**Published:** 2020-11-18

**Authors:** Iveta Zednikova, Eva Pazourkova, Sona Lassakova, Barbora Vesela, Marie Korabecna

**Affiliations:** 1 Department of Biology and Medical Genetics, First Faculty of Medicine, Charles University, Albertov 4, 128 00 Prague, Czech Republic; 2 Department of Biology and Medical Genetics, General University Hospital in Prague, Albertov 4, 128 00 Prague, Czech Republic; 3 Department of Nephrology, First Faculty of Medicine, Charles University and General University Hospital in Prague, U nemocnice 2, 128 08 Prague, Czech Republic

**Keywords:** Biological techniques, Genetics, Biomarkers, Molecular medicine

## Abstract

In families with X-linked recessive diseases, foetal sex is determined prenatally by detection of Y-chromosomal sequences in cell-free foetal DNA (cffDNA) in maternal plasma. The same procedure is used to confirm the cffDNA presence during non-invasive prenatal RhD incompatibility testing but there are no generally accepted markers for the detection of cffDNA fraction in female-foetus bearing pregnancies. We present a methodology allowing the detection of paternal X-chromosomal alleles on maternal background and the confirmation of female sex of the foetus by positive amplification signals. Using digital droplet PCR (ddPCR) we examined X-chromosomal INDEL (insertion/deletion) polymorphisms: rs2307932, rs16397, rs16637, rs3048996, rs16680 in buccal swabs of 50 females to obtain the population data. For all INDELs, we determined the limits of detection for each ddPCR assay. We examined the cffDNA from 63 pregnant women bearing Y-chromosome negative foetuses. The analysis with this set of INDELs led to informative results in 66.67% of examined female-foetus bearing pregnancies. Although the population data predicted higher informativity (74%) we provided the proof of principle of this methodology. We successfully applied this methodology in prenatal diagnostics in a family with Wiscott–Aldrich syndrome and in pregnancies tested for the risk of RhD incompatibility.

## Introduction

The discovery of cell-free foetal DNA (cffDNA) in maternal circulation in 1997^1^ opened a new era in non-invasive prenatal testing (NIPT). cffDNA in the maternal circulation serves as an alternative source of foetal genetic material to chorionic villi or amniocytes which are obtained using invasive techniques such as chorionic villus sampling or amniocentesis. These methods have a risk of miscarriage 1–2%, cause higher discomfort of patients, and can be applied during only certain time windows during pregnancy^2^.

cffDNA is released into the maternal blood stream due to the continuous placenta remodelling which involves the apoptotic events^3,4^. The apoptotic origin of the cffDNA molecules contributes to their characteristic size distribution with a prominent 143‐bp peak^5^. The increase of cffDNA fraction in plasma of pregnant women in the successive trimesters (with means 8.3, 10.7 and 23.2%) was well documented^6^. Between the 10th and 21st week of gestation, it increases by 0.1% every week. After the 21st week, the increment is higher reaching almost 1% every week^7^. The cffDNA fraction may be influenced by pregnancy-related maternal diseases^8^ and maternal body weight^9^, it is higher in the pregnancies with foetal aneuploidies^8^ and twins^10^. The analysis of cffDNA fraction in maternal circulation based on next generation sequencing (NGS) was elaborated for non-invasive screening of foetal aneuploidies and chromosomal aberrations^11^. The correct determination of the cffDNA fraction is crucial for the proper interpretation of all approaches developed for NIPT of foetal aneuploidies and based on next generation sequencing (NGS). When reporting such analysis, the measurements of cffDNA fraction should be included due to the risk of false negative results when this fraction is very low^12^.

NIPT techniques independent of NGS are restricted to the identification of alleles which are present in the foetal genome but not in the maternal one—for example *RHD* (Rh blood group, D antigen) genotyping in RhD–negative mothers^13^, detection of mutant paternal alleles in families with monogenic disorders, such as achondroplasia^14^ or early onset primary dystonia^15^, and the exclusion of affected status in autosomal recessive disorders, such as cystic fibrosis^16^ or β-thalassemia^17^ in families where the parents have different mutations and therefore the paternal allele can be determined on the maternal background.

Non-invasive determination of foetal sex based on cffDNA analysis is offered to families affected with X-linked recessive disorders, such as Duchenne muscular dystrophy or haemophilia A and B. Determination of foetal sex is beneficial also in cases of congenital adrenal hyperplasia where it allows early therapy of female foetuses^18^. From methodological point of view, the determination of male foetal sex in such cases is straightforward because the detection of Y-chromosomal sequences on maternal background is relatively easy using quantitative polymerase chain reaction (qPCR) (e.g.,^1,6–10,13,19^) or digital droplet PCR (ddPCR)^18^. The routine practice employs these techniques, the female foetal sex is usually reported when no Y-chromosomal signals are found in cell-free DNA isolated from maternal plasma.

However, in the case of a female foetus, detection and quantification of cffDNA fraction is more challenging because there are no universal foetal markers. NGS methodologies are effective for assessing foetal fraction^20,21^ but they are expensive and dependent on complicated bioinformatic interpretation. The analysis of DNA sequences differentially methylated in placenta and maternal genome provided promising results^22,23^ with regard to the detection and quantitative characterization of cffDNA fraction independent of the foetal sex. Recently, this methodology is further elaborated and its application for detection of foetal aneuploidies is tested^24^. Examinations of single-nucleotide polymorphisms^25^ or short tandem repeats^26^ were also explored with the goal to find suitable tool for the detection of cffDNA fraction in maternal plasma. The panel of 10 insertion/deletion (INDEL) polymorphisms was analysed using quantitative PCR in association with prenatally determined paternal genotypes^27^. All these studies were performed using quantitative PCR, the digital PCR was used only in the study focused on the non-invasive confirmation of paternal mutations causing achondroplasia in foetus^14^.

The ability to confirm the presence of cffDNA in maternal plasma is especially important in the RhD incompatibility testing when the female foetus is RhD negative. In such cases, there are no positive signals amplified from any foetal targets and therefore the risk of false negative result is increased. Such pregnant women will not receive anti-RhD immunoglobulin and will be at risk of haemolytic disease of the newborn in subsequent pregnancies^28^. The meta-analysis focused on RhD incompatibility testing^29^ showed the highest false negative rates 0.35% (95% CI 0.15–0.82) when inconclusive results were excluded. The false negative results were mostly reported in the first trimester. *RHD* genotyping was evaluated as sufficiently accurate when performed later than in the 11th week of gestation^28^.

Non-invasive detections of RhD incompatibility and foetal sex are routinely performed at the Institute of Biology and Medical Genetics (1st Faculty of Medicine, Charles University and General University Hospital in Prague, Czech Republic) since 2008. Plasma of pregnant women at risk of RhD incompatibility or X-linked recessive disease of the foetus is analysed using qPCR technology. If the sequences originated from the Y chromosome or the *RHD* gene are detected in maternal plasma, the cffDNA fraction could be determined and its size estimated. Nevertheless, RhD negative female foetuses are determined just by the absence of these signals. Thus, implementation of an effective method that would clearly demonstrate the presence of the cffDNA fraction in these pregnancies seemed to be essential for our laboratory workflow. We decided to employ the analysis of INDEL polymorphisms localized along the X-chromosome in combination with the ddPCR to solve this issue.

The successful detection of the Y-chromosomal sequences in maternal plasma using ddPCR was demonstrated^18^. The paternal alleles of autosomal INDEL polymorphisms were detected in cffDNA fraction in maternal plasma using ddPCR in females having suitable homozygous genotypes^19^. The large set of INDEL polymorphisms as markers was also used during the analysis of NGS data with the goal to determine the cffDNA percentage for NIPT^30^. We selected five INDEL polymorphisms known as suitable markers in forensic genetics localized along the entire length of the X chromosome^31–33^.

To our best knowledge, we present the first study demonstrating the usefulness of ddPCR for examination of the X-chromosomal INDEL polymorphisms leading to the detection of paternal X chromosomal sequences in cffDNA in plasma of pregnant women bearing female foetuses.

## Results

### Sensitivity and specificity of assays, limit of blank (LOB) and limit of detection (LOD)

The linearity of all assays was explored using nine-points calibration curves (Table 1, Supplementary Fig. 1). The specificity of all assays was determined on the sets of samples not containing the target sequence detected by the tested TaqMan probe. The average false positive count of copies per sample (λFP) and the average false positive ratio of all negative samples (R_FP_) were calculated (Table 1, Supplementary Table 2).

**Table 1:** Population characteristics of selected INDEL polymorphisms, performance characteristics of single assays and comparison of the theoretical and the observed informativity. λFP – average false positive counts of copies per 1 µl of reaction, R_FP_ – average false positive ratio of all negative samples, H–W – Hardy–Weinberg, LOB – limit of blank given in copies per 1 µl of reaction, LOD – limit of detection given in copies per 1 µl of reaction, I – insertion allele, D – deletion allele.

Indel	rs2307932		rs16397		rs16637		rs3048996		rs16680	
	**I** (FAM)	**D** (HEX)	**I** (HEX)	**D** (FAM)	**I** (HEX)	**D** (FAM)	**I** (FAM)	**D** (HEX)	**I** (HEX)	**D** (FAM)
R^2^	0.969	0.972	0.978	0.989	0.981	0.982	0.968	0.943	0.990	0.984
λFP (copies/µl)	0.000	0.004	0.004	0.000	0.017	0. 005	0.010	0.009	0.002	0.000
R_FP_	0.00	4.89e^−5^	7.16e^−5^	0.00	3.00e^−4^	9.26e^−5^	1.50e^−3^	1.11e^−4^	2.13e^−5^	0.00
LOB (copies/µl)	0.032	0.050	0.030	0.034	0.060	0.000	0.033	0.066	0.000	0.000
LOD (copies/µl)	0.060	0.092	0.057	0.063	0,110	0.000	0.063	0.119	0.000	0.000
Population frequency	0.48	0.52	0.30	0.70	0.45	0.55	0.51	0.49	0.31	0.69
H–W equilibrium (p value)	0.1341		0.2617		0.8487		1.000		0.8461	
Expected informative pregnancies (%)	24.96		21.00		24.75		24.99		21.39	
Observed informative pregnancies (%)	19.05		33.33		23.81		22.22		25.39

**Figure 1:**
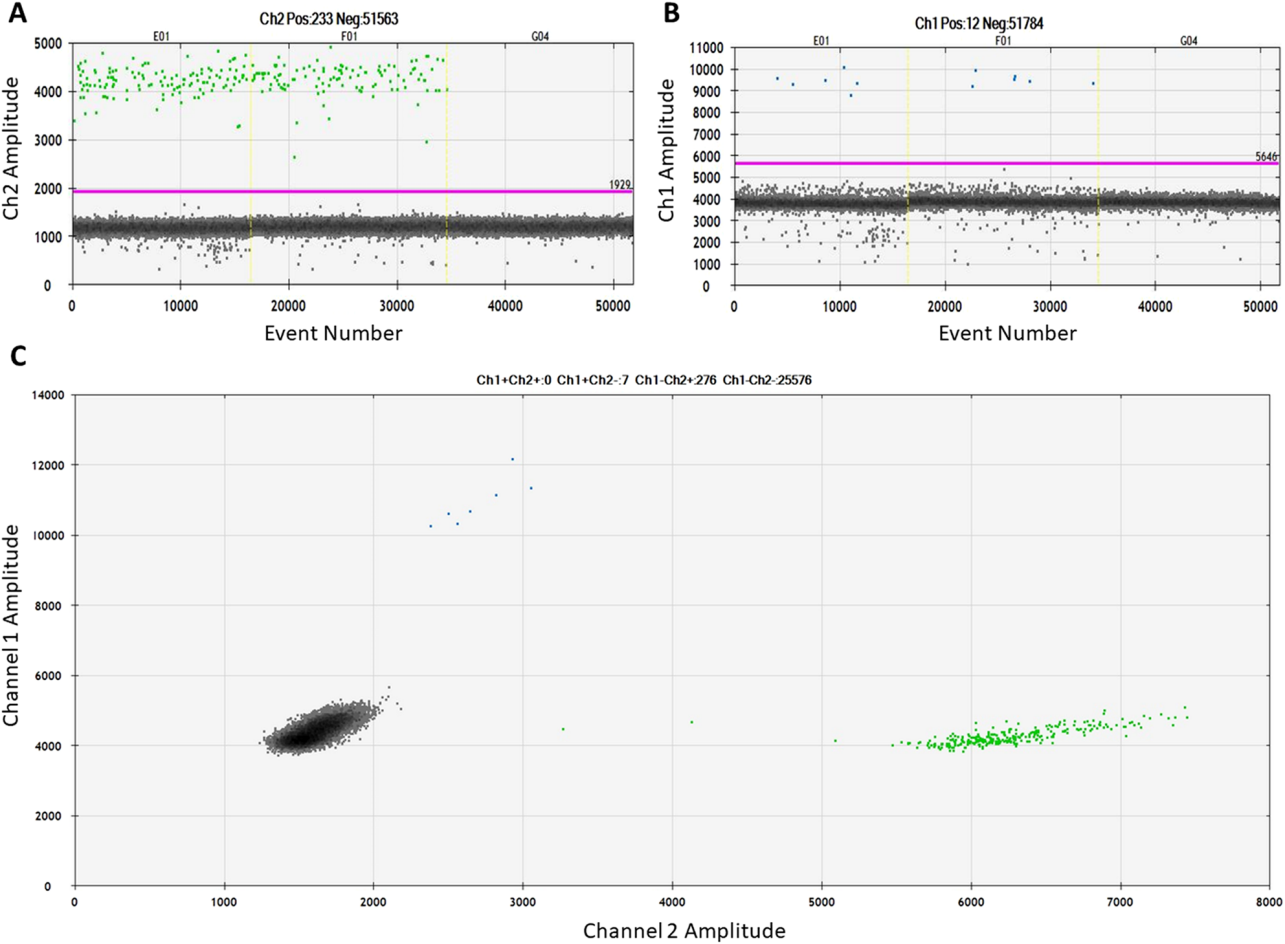
Examples of informative results obtained using the software QuantaSoft. **A** – Maternal fraction in sample no. 21, rs16637 (HEX), **B** – foetal fraction in sample no. 21, rs16637 (FAM). **C** – 2D plot for merged reactions for rs3048996 and Patient No. 54.

**Table 2:** Detection of paternal X-chromosomal alleles in informative clinical plasma samples. WG – week of gestation, No. – patient number, ND – not determined, _a_ – maternal genotype confirmed using buccal swab, values in bold – informative results, values in italic – under LOD.

WG	No.	rs2307932		rs16397		rs16637		rs3048996		rs16680		cffDNA fraction (%)
		Copies/ul		Copies/ul		Copies/ul		Copies/ul		Copies/ul		
		FAM	HEX	FAM	HEX	FAM	HEX	FAM	HEX	FAM	HEX	
8	57	0.00	13.30	**0.15**	16.35	18.35	0.00	21.35	**0.16**	10.35	**0.04**	1.80; 1.48; 0.77
9	14	0.00	6.20	7.75	**0.37**	5.20	4.65	**0.22**	8.05	6.50	0.00	8.71; 5.19
9	51	7.85	**0.40**	5.55	4.80	11.15	**0.17**	6.70	6.15	4.20	3.80	9.25; 2.95
10	32	3.63	3.13	7.93	0.00	10.6	**0.21**	4.40	4.23	6.17	0.00	3.81
10	52	7.15	**0.70**	5.20	5.55	10.95	0.00	4.55	6.15	6.40	0.00	16.57
10	53	0.00	1.65	0.00	2.80	1.75	1.80	3.55	**0.28**	2.25	0.00	12.78
10	58	0.00	7.65	**0.23**	9.30	5.40	6.05	12.60	0.00	**0.08**	6.80	5.67; 2.30
10	59	**0.34**	13.25	**0.61**	21.50	13.10	13.40	23.80	**0.80**	7.05	5.90	4.61; 5.40; 6.30
11	16	3.00	2.90	9.45	0.00	9.70	**0.16**	9.25	**0.33**	**0.09**	4.90	3.19; 6.66; 3.54
11	24	7.05	**0.16**	5.75	4.70	10.45	*0.07*	*0.035*	11.65	3.45	3.45	4.34
11	25	6.35	6.80	9.10	9.75	**0.80**	23.50	11.65	12.40	12.15	**0.16**	1.61; 2.57
11	29	3.00	2.70	9.60	0.00	**0.41**	11.23	5.20	5.70	5.00	**0.16**	6.80; 6.02
11	46	3.00	3.15	**0.25**	9.10	**0.30**	11.15	0.00	10.35	6.40	**0.11**	5.21; 5.11; 3.32
12	18	3.85	4.40	10.60	**0.24**	13.00	0.00	0.00	14.05	8.70	0.00	4.33
12	42	0.81	1.25	2.80	0.00	1.65	1.35	0.00	3.70	2.55	**0.11**	7.94
12	45	2.30	1.75	5.20	**0.14**	3.85	2.90	3.85	3.10	2.00	1.85	5.11
12	49	5.30	**0.13**	**0.27**	8.85	5.75	4.25	0.00	10.00	3.15	2.65	4.68; 5.75
14	62	4.15	5.40	18.55	15.90	17.40	22.85	17.40	23.95	24.50	**1.40**	10.26
15	26	0.00	5.00	9.15	**0.60**	0.00	8.80	3.20	5.20	2.60	2.10	11.59
15	27	3.67	3.17	0.00	8.00	**0.25**	11.30	4.10	4.53	7.40	**0.18**	4.24; 4.64 (eight replicates analysed)
16	11_a_	83.33	**1.30**	171.33	**2.63**	ND	ND	181.33	**3.9**	ND	ND	3.03; 2.98;4.12
17	2_a_	ND	ND	8.00	**0.10**	**0.06**	10.10	ND	ND	6.07	*0.03*	2.44; 1.17
17	39_a_	ND	ND	3.80	**0.15**	ND	ND	ND	ND	2.73	0.00	7.32
17	21	3.1	2.6	6.4	**0.18**	**0.41**	7.95	4.25	3.55	5.65	0.00	5.33; 9.35
19	5_a_	ND	ND	5.67	**0.23**	**0.09**	6.73	ND	ND	ND	ND	7.50; 2.60
19	12_a_	ND	ND	6.80	**0.12**	ND	ND	**0.35**	7.47	5.4	**0.10**	3.87; 8.57; 3.57
20	9_a_	ND	ND	11.93	**0.24**	ND	ND	ND	ND	8.50	**0.04**	3.87; 0.93
20	10_a_	**0.36**	10.63	19.37	0.00	0.00	20.20	ND	ND	**0.08**	13.67	6.34; 1.16
20	34_a_	ND	ND	ND	ND	5.48	0.00	**0.25**	6.60	ND	ND	7.04
20	61	0.90	0.95	6.85	**0.14**	0.00	7.60	2.70	3.35	4.65	**0.05**	3.93; 2.11
21	22	**0.95**	11.70	8.95	9.10	11.05	6.70	20.60	0.00	5.55	3.80	13.97
23	6_a_	**0.59**	48.33	92.00	0.00	ND	ND	**1.43**	82.00	ND	ND	2.38; 3.37
23	8_a_	ND	ND	**0.28**	5.86	ND	ND	ND	ND	**0.19**	4.73	8.72; 7.44
24	38_a_	ND	ND	4.70	**0.31**	5.07	**0.25**	ND	ND	ND	ND	11.65; 8.77
24	63	1.05	0.55	6.60	**0.17**	**0.15**	7.40	3.65	2.34	4.25	0.00	4.90; 3.90
25	60	0.78	0.70	5.85	0.00	6.35	**0.37**	5.90	**0.27**	4.95	0.00	10.18; 8.39
30	4 _a_	*0.03*	3.50	6.47	0.00	7.07	0.00	**0.53**	5.73	ND	ND	15.61
33	13_a_	3.10	**0.26**	ND	ND	ND	ND	5.67	**1.00**	ND	ND	14.36; 26.07
36	7_a_	12.20	**1.77**	23.20	0.00	**3.70**	20.33	ND	ND	**1.83**	10.20	22.49; 27.77; 26.41
ND	48	6.80	**0.25**	4.45	5.35	0.00	11.1	9.85	**0.40**	5.9	**0.04**	6.85; 7.51; 1.33
ND	50	6.65	4.65	13.40	0.00	16.35	**0.25**	7.65	9.05	10.15	0.00	2.97
ND	54	0.00	6.45	10.05	**0.13**	6.50	5.75	**0.32**	12.65	8.10	0.00	2.52; 4.82

Both limit of blank (LOB) and limit of detection (LOD) for all INDELs in both channels were determined. The results are summarized in Table 1 and raw data are presented in Supplementary Table 2.

The criteria for the reliable detection of cffDNA fraction in plasma samples of pregnant patients were defined: The number of all accepted droplets in the sample must be higher than 20,000, the value characterising the cffDNA fraction given in copies/µl in reaction must be higher than LOD, minor fraction should not exceed 20% of the plasma cfDNA to be regarded as the fraction of foetal origin. The only exceptions are represented by the rare patients in the last weeks of pregnancy (patients no. 7 and 13 in Table 2 and Supplementary Table 3).

**Table 3:** Sequences of primers and probes for the analysed INDEL polymorphisms.

rs2307932	Sequence	Labelling	Amplicon length (bp)
Forward primer	ACTTCCAACTAAGTTAATCTCT		
Reverse primer	TTCCAAAATTTCTCAAAGGC		
Probe for insertion variant	AGTCTCAGAATCTTataaTAATATCTTTT	FAM	204
Probe for deletion variant	AGTCTCAGAATCTTaTAATATCTTTTTT	HEX	198
**rs16397**			
Forward primer	TGCCAAAGCATATAAAATGG		
Reverse primer	TGATGGTGTCTTGTATTTCT		
Probe for insertion variant	AAGGGTATGAAgtggTGAC	HEX	148
Probe for deletion variant	ACAAGGGTATGAAgTGACTAT	FAM	142
**rs16637**			
Forward primer	TGATATGAAGTCTGGTATTGG		
Reverse primer	TTATTTCCTCACTTCTCCAC		
Probe for insertion variant	ACTcaaccaatgGGGC	HEX	158
Probe for deletion variant	AAATACTgGGGCTGTTTAAC	FAM	142
**rs3048996**			
Forward primer	GACCCACGGTGTTGAAT		
Reverse primer	AGATAGACAGGAGATGAGTG		
Probe for insertion variant	ATTTGCTTatcaTCCATCCAG	FAM	136
Probe for deletion variant	TTGCTTaTCCATCCAGCC	HEX	130
**rs16680**			
Forward primer	AGAGAAGGCATCTTCTATATG		
Reverse primer	ATCTGTGGGAACCCTATTAT		
Probe for insertion variant	TTAACCAAGtacaACAACTGT	HEX	188
Probe for deletion variant	CACTTAACCAAGtACAACTGT	FAM	182

### Population characteristics of selected INDEL polymorphisms

We determined the genotypes in 50 unrelated females of the Czech origin (Supplementary Table 1). We used these data to detect if the observed frequencies of genotypes are in agreement with Hardy Weinberg equilibrium in our population. We found non-significant differences (Table 1). The population data served us for the calculation of the percentage of potentially informative pregnancies for each INDEL polymorphism (Table 1). The proportion of the pregnancies which will be non-informative for all INDELs in the set was determined as 26%. In other words, the examination of this set of INDEL polymorphism should be theoretically informative in 74% of pregnancies according to our population data.

### Examination of plasma samples of pregnant women

Only plasma samples which were tested routinely and reported as negative for Y-chromosomal sequences were analysed. The informative results are listed in Table 2. The patients with maternal genotypes confirmed by examination of buccal swabs are marked in this table. Selected examples of informative analyses are presented in Fig. 1.

The percentages of informative pregnancies theoretically awaited and practically detected in this pilot cohort are compared in Table 1. The raw data achieved in all examined patients are presented in Supplementary Table 3. We found 42 (66.67%) informative pregnancies in which the paternal X-chromosomal allele was detected on maternal background. With regard to the informative samples, 14 (33.33%) patients were informative for only one INDEL, 20 (47.62%) for two INDELs, and 8 (19.05%) for three INDELs (Table 2, Supplementary Table 3). The cffDNA fraction was calculated using the results for each informative INDEL as described in Materials and methods.

### Case reports

In August 2014, a 30-year-old woman came for non-invasive prenatal diagnosis of foetal sex from the venous blood of the mother. She was a carrier of the X-linked disease called Wiskott-Aldrich syndrome (WAS) and now she was at her eleventh week of pregnancy. Her gestational age based on a first-trimester obstetric ultrasonography was 10 + 0.

WAS is a rare X-linked recessive disease. It is characterized by eczema, thrombocytopenia with bleeding complications and immune deficiency with recurrent bacterial infections. The treatment is currently based on correcting the symptoms and the only causal therapy could be the hematopoietic stem cell transplantation or gene therapy in the future^34^.

Non-invasive prenatal testing for foetal sex is a method routinely performed at our department. The real-time PCR detection of the Y-chromosomal *DYS 14* sequence is performed usually in seven replicates.

In this case, the *DYS 14* sequence was not detected in the usual range of C_t_ values (C_t_ values under 35) in any replicate. However, in all replicates there were low non-specific signals with late C_t_ values about 38–39. Another blood sample from this patient was taken two weeks later. The exactly identical results were obtained. There was no possibility to verify whether these late signals were really non-specific and thus the foetus was female as it should be expected from these results. The woman had to undergo invasive CVS testing with 46, XX result. She delivered a healthy daughter in term.

In October 2019, another carrier of the Wiskott-Aldrich syndrome was indicated for NIPT of foetal sex. The patient was 25 year-old woman and she was the full sister of the woman previously tested in 2014. Her gestational age was 10 + 4 based on the first-trimester obstetric ultrasonography. As usually cell-free DNA was extracted from the plasma sample and it was analysed by the qPCR method. No *DYS 14* sequence in any replicate was determined.

To confirm the female sex of the foetus, the set of five INDEL polymorphisms on the X chromosome was genotyped using ddPCR according to the procedure described in Materials and Methods. The minor foetal fraction was detected in two out of five INDELs analysed and the presence of the paternal X-chromosome in the foetal genome was proven (the Patient No. 29 in Table 2 and Supplementary Table 3). The invasive prenatal testing intended by this patient if the NIPT would provide negative results for Y-chromosomal sequences was avoided.

The ddPCR analysis of this set of five INDELs was implemented in the workflow of our diagnostic laboratory for non-invasive confirmation of female foetal sex in pregnancies of RhD negative women with foetuses reported as RhD negative ones. Typical successful examples of this approach are represented by patients 27, 38 or 46 in Table 2.

## Discussion

We demonstrate the usefulness of ddPCR genotyping of selected INDEL polymorphisms for the paternal X chromosome detection in cffDNA in maternal plasma for non-invasive prenatal diagnostics. With regard to the limited number of analysed INDELs with carefully determined population characteristics and relatively small size of our cohort of pregnant patients, the study could serve as a proof of principle.

The forensic applications of the X-chromosomal INDELs are well known, the density of suitable polymorphisms of this type on the chromosome X^30–33^ allows the selection of further markers to enlarge this original set and to reach higher informativity of this methodological approach. It is necessary to keep in mind that the clinical performance of each set of markers will be highly dependent on the population frequencies of their allelic variants therefore the optimal sets of markers may differ among different populations.

Our workflow allows the detection of cffDNA fraction in female foetus bearing pregnancies and provides the evidence that the paternal X-chromosomal sequences are present in this fraction. The exact determination of the cffDNA fraction size is difficult when only three replicates per INDEL are used for ddPCR analysis. According to our pilot experiment, it seems that an increased number of replicates could lead to the detection of higher number of signals for cffDNA fraction and contribute to the accuracy of the technique in terms of correct determination of this fraction size with nearly identical values for samples analysed using more informative INDELs. The higher number of positive signals is necessary for correct quantification of the cffDNA fraction size. The numbers achieved by us are above the LODs and therefore sufficient to detect this fraction but too low to serve for its accurate quantification. To overcome this limitation, it would be necessary to isolate the cfDNA from larger volume of plasma and to concentrate the eluated samples or run more technical replicates as we demonstrate in the selected case. When the number of positive partitions that should be quantified is lower than 1000 as it is in foetal cfDNA fractions detected by us, the Poisson model used for copy number estimation will have a coefficient of variation of at least 4% and this fact will represent one of the main factors contributing to the measurement imprecision^35^. We focused on the first trimester pregnancies as the lowest fetal cfDNA fraction could be supposed in this period of pregnancy. When the results obtained for the pregnancies in the gestational weeks eight to twelve (n = 25) were compared with those being in higher gestational weeks (n = 34) for each INDEL separately using chí-square test, no significant differences were found. The LODs of all assays were low enough to detect foetal fractions therefore we evaluated population structure and small size of our cohort as the main factors contributing to the differences between predicted and observed values.

We introduced the new methodological approach giving us the possibility to prove non-invasively the female foetal sex by positive PCR signals not only by the absence of any amplification. After further careful elaboration (the addition of further INDEL polymorphisms and increase in number of ddPCR replicates for each INDEL) in association with further technical development in the field of digital PCR, this methodology has the potential to achieve nearly 100% sensitivity. The technical development is desired particularly with regard to the low concentration of cffDNA in plasma samples and the necessity to minimize the sample consumption.

The previous studies dealing with foetal cfDNA detection and qualitative analysis were mostly based on quantitative PCR^22,25–27^. The hypermethylated *RASSF1* sequences were reported as a universal marker for the foetal cfDNA detection and quantification^22^ but the authors were not able to detect the foetal fraction in four out of nineteen RhD negative pregnant patients using this methodology. The higher ddPCR tolerance of suboptimal amplification efficiency could reduce the risk of false negative classification. The only study^14^ employed ddPCR to detect the foetal cfDNA fraction using hypermethylated *RASSF1* sequences in the third trimester pregnancies and concluded that only foetal cfDNA fractions higher than 4% were detectable. The foetal cfDNA fraction is probably not completely hypermethylated therefore the digestion with methylation sensitive enzyme can reduce its size. The results presented in Table 2 document that our ddPCR based methodology is able to detect and confirm the presence of fetal cfDNA fraction lower than 4%.

Regarding the cited results, the development of new methodological approaches for a universal foetal cfDNA fraction detection and quantification seems to be highly desirable.

We demonstrated that our method in its current state helped us to avoid the intended invasive prenatal testing in a family with Wiskott–Aldrich syndrome and to prevent the reporting of false negative results of non-invasive foetal *RHD* genotyping in RhD negative pregnant women.

## Methods

### Subjects

All samples included in our study were obtained from pregnant women who underwent routine prenatal diagnostic procedure for foetal sex determination or *RHD* genotyping in cooperation with Department of Obstetrics and Gynaecology of the First Faculty of Medicine, Charles University and General University Hospital in Prague. The written informed consents were obtained from all participants. The study was conducted in accordance with the Declaration of Helsinki and approved by the Ethical Committee of the First Medical Faculty of Charles University and General Faculty Hospital in Prague.

A total of 50 buccal swabs which were collected for the detection of eventual abnormal *RHD* genotype of pregnant women were included in the initial study phase. These samples were used for the population study and for the selection of the potentially informative INDEL polymorphisms (homozygous for one of the alleles) for the following analysis of the relevant plasma sample.

A total of 63 plasma samples of pregnant women were included in the subsequent study phase. All these plasma samples were analysed within routine diagnostic procedure as negative for Y-chromosomal sequences. Thirteen samples were paired to the buccal swab samples examined in the study phase focused on population characteristics of selected INDELs.

### Preparation of plasma samples

Peripheral blood samples were collected by venipuncture using Vacutainer tubes with EDTA to prevent coagulation. Tubes were stored at 4 °C and processed within 6 h after sampling. Two-steps centrifugation was performed to obtain plasma from peripheral blood samples: 2600 g for 10 min at 10 °C, and 14,500 g for 10 min at room temperature. Plasma samples were finally frozen to − 20 °C.

### DNA isolation

For purification of cell-free DNA from 1 ml of plasma, the automated MagNA Pure Compact System with MagNA Pure Compact Nucleic Acid Isolation Kit I—Large Volume (Roche Diagnostics, Germany) was used. Finally, cfDNA was eluted in 50 or 100 μl of supplied elution buffer and processed immediately.

Genomic DNA was collected using buccal swabs (Copan Innovation, Italy). DNA was then isolated using QIAamp Mini Kit (Qiagen, USA) according to manufacturer’s instruction. Purified DNA was stored at − 20 °C. Before the use as a template for ddPCR reaction, genomic DNA was enzymatically digested using HaeIII restriction endonuclease (Promega, USA) according to the manufacturer's instructions.

### Digital droplet PCR

The INDELs rs2307932; rs16397; rs16637; rs3048996; rs16680 were genotyped. Custom assays (Primers + TaqMan hydrolysis probe, two assays for each INDEL) specific for ddPCR were designed by Bio-Rad (USA). For each INDEL, one of the variants (insertion or deletion) was labelled with FAM fluorophore and the opposite variant with HEX fluorophore (sequences of primers and probes together with their labelling and the lengths of amplicons are listed in Table 3).

At first, all ddPCR reactions were optimized by temperature gradient (48–65 °C) to choose appropriate annealing temperature. The most suitable ramp rate was tested as well. The composition of the reaction mixture was as follows: 10 μl ddPCR Supermix for Probes (Bio-Rad), 1 μl Assay 1 (FAM labelled; Bio-Rad), 1 μl Assay 2 (HEX labelled; Bio-Rad), 3 μl water for injections, 5 μl DNA.

20 μl of prepared reaction mixture was then mixed with 70 μl of Droplet Generation Oil for Probes (Bio-Rad) using QX100 Droplet Generator (Bio-Rad) to form an emulsion of droplets. Amplification of target sequences was performed using T100 Thermal Cycler (Bio-Rad). The following thermal profile was applied: 95 °C for 10 min, 40 cycles of 94 °C for 30 s and 50 °C for 1 min, one hold 98 °C for 10 min and final holding 12 °C.

Ramp rates were set to 3 °C/s. All ddPCR reactions were run in duplicates, triplicates or quadruplicates according to the respective elution volume and number of tested INDELs. No template control (NTC) was always included. After ddPCR amplification, samples were loaded into the QX100 Droplet Reader (Bio-Rad). Final evaluation was performed by QuantaSoft Software version 1.6.6.0320 (Bio-Rad). The linearity of all assays was explored using dilution series in the range 1.000–0.003 ng/µl of sample.

### Limit of blank (LOB), limit of detection (LOD) and false positivity (FP) determination

LoB was determined for each assay using the set of samples without the tested target (Supplementary Table 2) and calculated according the formula: LOB = mean_blank_ + 1,645 (SD_blank_)^36^. LOD was calculated as: LOD = mean_blank_ + 3,29 (SD_blank_)^37^. Specificity of all assays was determined on the sets of samples not containing the target sequence detected by the tested TaqMan probe represented by the genomic DNA isolated from buccal swabs of homozygous females (Supplementary Table 2). The average false positive count of copies per sample (λFP) and the average false positive ratio of all negative samples (R_FP_) were calculated according to the literature^38^.

### Population study

To determine the allelic frequencies in Czech population, we performed genotyping of five selected INDEL polymorphisms on buccal swab samples of 50 unrelated women using ddPCR. The population data served for the calculation of the percentage of potentially informative pregnancies (*p*^2^ * *q* + *q*^2^ * *p*) for each INDEL and for the estimation of the proportion of pregnancies which could be non-informative for all INDELs in the set. The fractions of non-informative pregnancies for each INDEL were calculated as 1 − (*p*^2^ * *q* + *q*^2^ * *p*). All these fractions were then multiplied to calculate the fraction of non-informative pregnancies for the whole set of INDELs.

### Interpretation of results obtained on plasma samples of pregnant women

Plasma samples of pregnant women which have been processed at our institute within routine diagnostic procedures in the last three years were reviewed. Samples which were negative for both Y-chromosomal and RHD gene sequences were subjected to INDEL genotyping.

In the first step, only plasma samples with maternal INDEL genotypes known from the analysis of buccal swabs were further processed. Thirteen potentially informative (homozygous in at least one INDEL) women were selected for the subsequent analysis of relevant plasma samples. Only such polymorphisms in which the pregnant woman was homozygous and the different paternally inherited allele could be therefore potentially detected in cffDNA fraction in plasma were analysed using ddPCR technology. In the next phase, all plasma samples without prior determination of maternal INDEL genotypes were examined. The differentiation between heterozygous mother and homozygous mother with the different paternal allele in cffDNA fraction was possible due to the absolute quantification of both allelic variants provided by ddPCR.

### Determination of cffDNA fraction

The paternally inherited allele in cffDNA is detectable only if pregnant woman is homozygous for the opposite allelic variant. The foetus is then a heterozygote and the detected cffDNA represents only a half of the total cffDNA fraction. The percentage of cffDNA in the total cell-free DNA in plasma of the pregnant woman (cfDNA) was therefore calculated as follows: *cffDNA fraction* (%) = [100/(*cfDNA* + *cffDNA*)/*cffDNA*] * 2.

In one case of discrepancies in determined cffDNA fractions by two different INDEL polymorphisms, the sample was re-analysed. For more accurate quantification, eight replicates instead of three ones were successfully examined (Patient No. 27 in Table 2 and Supplementary Table 3).

### Statistical analysis

We employed QuickCalc (https://www.graphpad.com/quickcalcs/chisquared1) for the chi square tests and STATISTICA (StatSoft, USA) for the interpretation of mixture analyses. We used significance level α = 0.05 for all comparisons.

## Supplementary information

Supplementary Table 1.

Supplementary Table 2.

Supplementary Table 3.

Supplementary Figure 1.
