# Supplementary material for: Detection of cell-free foetal DNA fraction in female-foetus bearing pregnancies using X-chromosomal insertion/deletion polymorphisms examined by digital droplet PCR
Source: Sci Rep. 2020 Nov 18;10:20036. doi: 10.1038/s41598-020-77084-0 (PMC7676229; doi:10.1038/s41598-020-77084-0)
Supplement: Supplementary file 1 — Supplementary Table 1. [file 41598_2020_77084_MOESM1_ESM.pdf]

Table S1 Population data for selected INDEL polymorphisms, (n=50 females), + insertion allele, - deletion allele

INDEL	Homozygotes ++ (%)	Heterozygotes +- (%)	Homozygotes -- (%)
**rs2307932**	16	64	20
**rs16397**	14	32	54
**rs16637**	18	54	28
**rs3048996**	26	50	24
**rs16680**	8	46	46
