# Supplementary material for: Detection of cell-free foetal DNA fraction in female-foetus bearing pregnancies using X-chromosomal insertion/deletion polymorphisms examined by digital droplet PCR
Source: Sci Rep. 2020 Nov 18;10:20036. doi: 10.1038/s41598-020-77084-0 (PMC7676229; doi:10.1038/s41598-020-77084-0)
Supplement: Supplementary file 4 — Supplementary Figure 1. [file 41598_2020_77084_MOESM4_ESM.pdf]

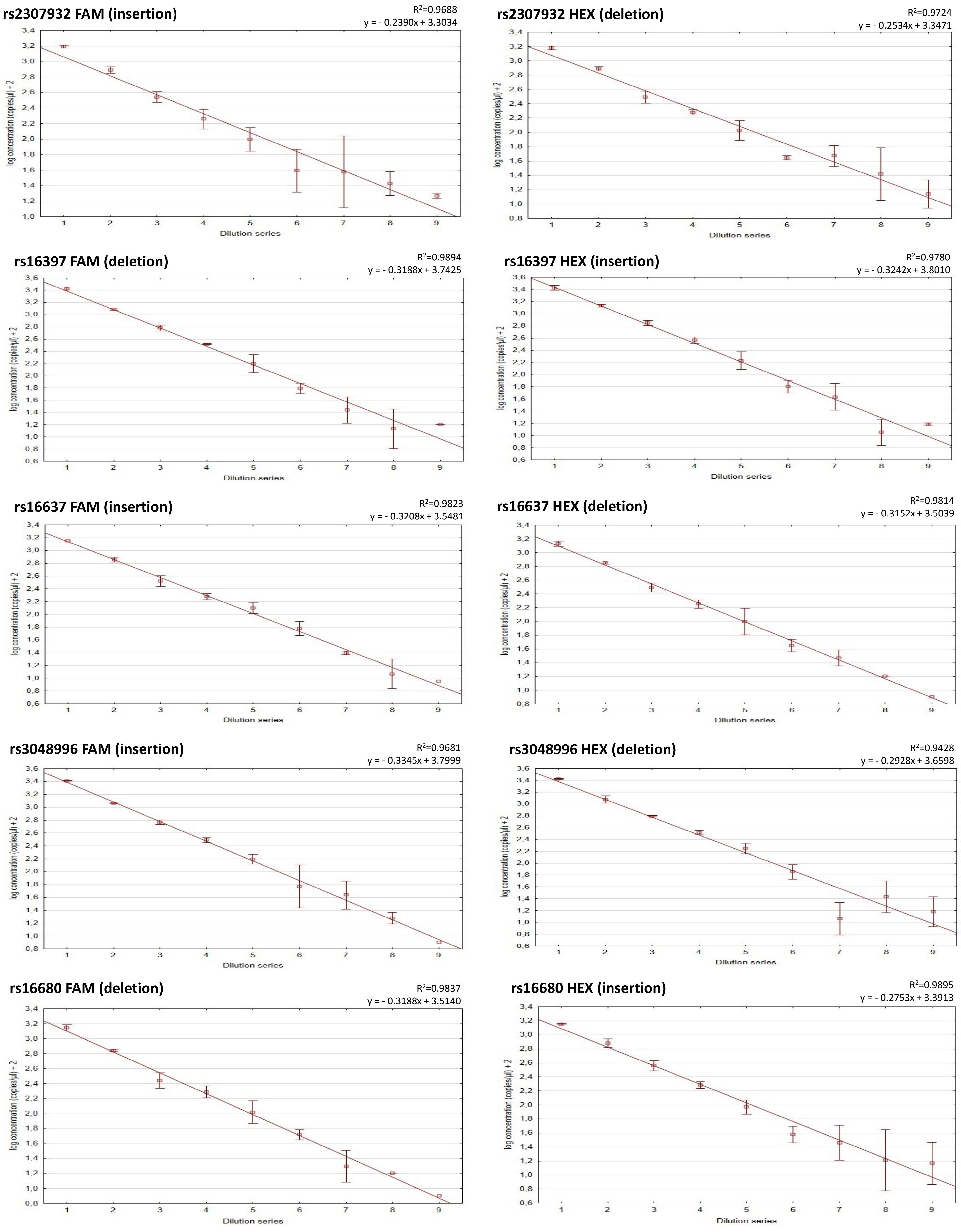

Supplementary Figure 1. Standard curves for all assays. Dilution series in the range 1.000 to 0.003 ng/µl of sample, mean values (squares) obtained from reactions run in triplicates were used for construction of these curves. Standard deviations of the mean are represented by error bars.
